# Sausage digit: Isolated tuberculous tenosynovitis of the middle finger

**DOI:** 10.1016/j.idcr.2022.e01438

**Published:** 2022-02-01

**Authors:** Wael Goravey, Muna Al Maslamani, Mahir Petkar, Adham Ammar, Gawahir A. Ali

**Affiliations:** a Department of Infectious Diseases, Communicable Diseases Centre Hamad Medical Corporation, Doha, Qatar; b Department of Laboratory Medicine and Pathology Hamad Medical Corporation, Doha, Qatar

**Keywords:** Tuberculous tenosynovitis, Middle finger, Synovectomy, Extra-pulmonary tuberculosis

## Abstract

Isolated tuberculous tenosynovitis is a rare form of extra-pulmonary tuberculosis that frequently eludes assessment and constitutes diagnostic challenges

A 52-year-old right-handed male, a manual worker, was seen in our clinic complaining of a four-month history of swelling in his right middle finger associated with discomfort. He had no history of recent trauma or constitutional symptoms. Examination findings were confined to a non-tender swollen in the right middle finger extending proximally to the wrist, suggesting tenosynovitis with limitation of the handgrip. Laboratory tests were within normal limits including a C-reactive protein level of 3 mg/L (0−5). MRI of the hand showed tenosynovitis of the middle finger extending to the flexor retinaculum with no osteomyelitis (Fig. 1A and B). The differential diagnoses included chronic infection, granulomatous disease, or inflammatory conditions. An open biopsy showed thick, jelly-like yellow tissue extending along the flexor tendons of the middle finger to the carpal tunnel. An extended synovectomy was performed. Necrotizing granulomatous inflammation was observed in the biopsy (Fig. 2) and GeneXpert MTB/RIF was positive with a negative rifampin resistance gene. Chest X-ray demonstrated no pulmonary involvement. Subsequently, he was started on 9 months of tuberculosis (TB) therapy (An intensive phase of 2 months of isoniazid (INH), rifampin (RIF), pyrazinamide (PZA), and ethambutol (EMB) followed by a continuation phase of 7 months of INH and RIF) with significant recovery of the handgrip. The patient had no recurrence one year into follow-up.

**Fig. 1:**
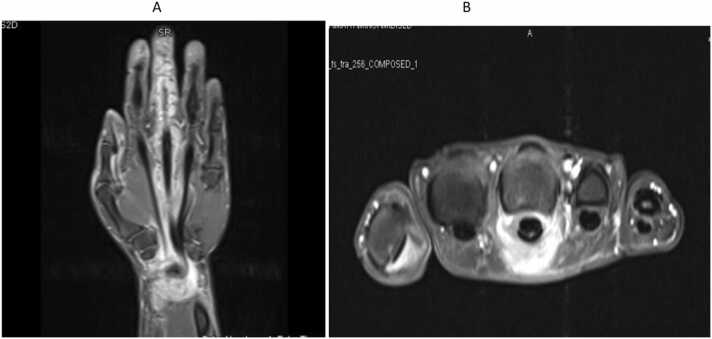
A and B MRI of the right hand demonstrating right middle finger tenosynovitis extending to the carpel tunnel.

**Fig. 2:**
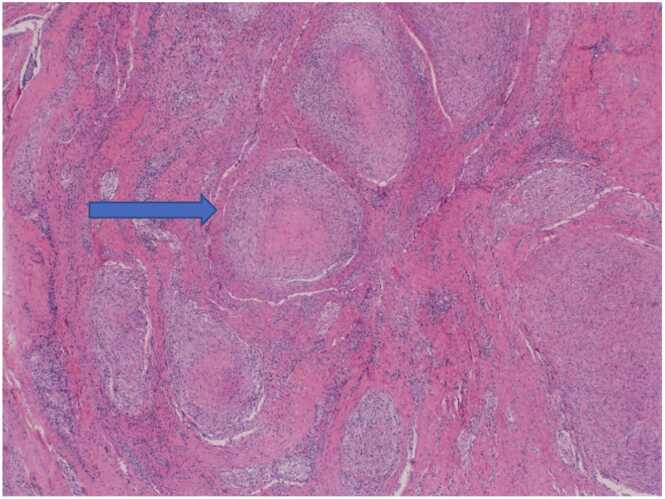
Histopathological examination of synovectomy tissues (Low power view) showing multiple necrotizing granulomatous inflammations, arrowed (H and E x 4).

Extra-pulmonary TB can affect any viable tissues including tendons of the finger, thereby raising diagnostic challenges [1]. Tuberculous tenosynovitis, a rare form of hand TB, accounts for 5% of all musculoskeletal TB cases [2]. Older males are infected more than young females, while the flexor tendons and dominant hand are more frequently affected [2]. Various clinical presentations exist, including compound palmar ganglion, sausage digit, and carpal tunnel syndrome [1]. It can imitate many infectious and noninfectious conditions leading to devastating consequences [3]. MRI features can suggest the diagnosis, but GeneXpert MTB/RIF clinch the diagnosis promptly pending the histopathological examination to rule out other potential causes [1], [4]. The mainstay of management is standard TB therapy for 6–9 months [2]. However, adjuvant operative treatment is required for advanced disease or failed medical therapy [1].

## Funding

No funding was received towards the publication.

## CRediT authorship contribution statement

**Wael Goravey:** Clinical management, contribute to data acquisition, manuscript preparation and final proof reading. **Muna A. Muslamani:** Supervised all the aspects and contributed to final manuscript editing. **Mahir Petkar:** Contributed to data acquisition and histopathology reports. **Adham Ammar:** Contributed to data acquisition and histopathology reports. **Gawahir. A. Ali:** Clinical management, data acquisition and manuscript writing.

## Ethical approval

Ethics approval and permission was obtained to publish the case reports from the institutional review board which is in line with international standards.

## Consent

Written informed consent was obtained from the patient to publish this report in accordance with the journal's patient consent policy.

## Conflict of interest

The authors declare that they have no competing interests.

## Data Availability

The authors confirm that the datasets supporting the findings of this case are available from the corresponding author upon request.
